# Study on Morphology and Chemical States of Surface Active Layer of Th-W Cathode

**DOI:** 10.3390/ma15082726

**Published:** 2022-04-07

**Authors:** Yin Cheng, Yuan Sun, Yizhou Zhou, Shiyang Wang, Jie Meng, Nan Cao, Wanpeng Shi

**Affiliations:** 1 Shi-Changxu Innovation Center for Advanced Materials, Institute of Metal Research, Chinese Academy of Sciences, Shenyang 110016, China; ycheng19b@imr.ac.cn (Y.C.); sywang16b@imr.ac.cn (S.W.); jmeng@imr.ac.cn (J.M.); 2 School of Materials Science and Engineering, University of Science and Technology of China, Shenyang 110016, China; 3 Liaoning Zhongke Boyan Technology Co., Shenyang 113122, China; lnzkby@163.com; 4 Foshan Huizhen Technology Co., Foshan 528251, China; foshanhuizhen@163.com

**Keywords:** thorium, organization, surface, active layer, chemical states

## Abstract

The surface morphology and chemical states of W-2%ThO_2_ thermionic cathode during vacuum high-temperature treatment were investigated in this research. The W-2%ThO_2_ thermionic cathode was prepared by a solid-liquid doping method combined with high-temperature sintering. The morphology and distribution of thorium oxide were observed using a transmission electron microscope and scanning electron microscope. The chemical states of elements at different temperatures were analyzed by X-ray photoelectron spectroscopy. Results indicate that the surface morphology and chemical form of the alloy evolve with the increase of temperature. The matrix had a lamellar structure at low temperatures, and the surface was relatively flat. The samples were heated to 500 °C, 1100 °C, and 1300 °C for 1 h. During the heating process, thorium oxide changed from granular to spherical, and the matrix was recrystallized. As the heating temperature rises, diffusion channels appear inside the cathode. As the temperature increases, the high-priced tungsten gradually decreases, and the zero-valent tungsten content increases. The adsorbed oxygen left the cathode surface, and the lattice oxygen increased. The surface oxygen content decreased, and the thorium and tungsten content increased.

## 1. Introduction

The magnetron is an electric vacuum device used to generate microwave energy. It is essentially a diode placed in a constant magnetic field [1]. The electrons in the tube interact with the high-frequency electromagnetic field under the control of the constant magnetic field and the constant electric field perpendicular to each other and convert the energy obtained from the continuous electric field into microwave energy, thereby achieving the purpose of generating microwave energy [2]. As the core component of this electronic device, the performance of the hot cathode directly determines the functional characteristics and service life of the electronic component [3,4]. ThO_2_ additions are commonly used in tungsten-based electrodes due to their excellent electron emissivity, higher strength, and machinability [5,6]. Since thorium is radioactive, it is desirable to replace classical thoriated tungsten electrodes with other types of electrodes whenever it is possible [7,8,9]. Therefore, it is essential to understand the working principle of the thoriated tungsten cathode to provide a theoretical basis for the development of new cathode materials.

Thorium cathode belongs to the oxide cathode, formed by adding ThO_2_ to the tungsten matrix [10,11]. Reports in the literature on thoriated tungsten cathode materials have focused mainly on the internal organization, neglecting the surface structure and the variation of the elements themselves. In fact, for hot electron-emitting materials, electron emission occurs mainly within a certain depth of the surface layer, and changes in the organization and composition within this range have an essential influence on performance. The cathode will be accompanied by changes in the valence and content of the element during use [12,13]. The change of part valence in the cathode will affect the physical and chemical properties of the material [14]. Therefore, it has guiding significance for performance improvement to carry out the judgment and analysis of the valence state of the cathode material. For this type of electron emission material, the surface-active component has a strong influence on the emission performance of the hot cathode [15,16]. The cathode material interacts with the surrounding environment through its surface. Changes in the physical and chemical composition of these surfaces determine the nature of the interaction [17,18].

With the rapid development of microwave vacuum devices, increasing the electron emission current density at a specific operating temperature is one of the main objectives of improving the performance of vacuum electron tube hot cathodes. Many reports have focused on the cathode composition optimization and cathode preparation methods. Still, there are few studies on the surface morphology and chemical states of the Th-W cathode in practical work. In this paper, the morphology, composition distribution, and chemical states change of the surface-active layer was observed at 500 °C to 1300 °C. Then, the zero-field current emission density and work function were measured using cathode emission performance test benches, which provided a reference for practical application.

## 2. Experiment Methods

A solid-liquid doping method was used to prepare a tungsten-based material containing 2% thorium oxide. Thorium is added at 2%, and the remainder is blue tungsten. Put thorium nitrate into deionized water and stir for 10 min to obtain a clear thorium nitrate solution. Add blue tungsten, go for 30 min, turn on steam for heating, and dry while stirring. Turn on the cooling water for cooling for 1 h, and then discharge the material. If there is hardensd, sieve with an 80-mesh screen to obtain thorium-doped blue tungsten. Hydrogen gas is used for reduction; the temperature is 680/730/780/820 °C, the boat capacity: is 200 g, the hydrogen flow rate: is 20 L/min, the boat pushing speed: is 20 min/each ship. Followed by pressing and sintering, holding pressure for 120 s, pre-sintering temperature are: 1350 °C, speed 30 min per boat. The billet sample is subjected to rotary forging and wire drawing. The parameters of the rotary forging hammer (Institute of Metal Research, Chinese Academy of Sciences, Shenyang, China) are: 1350 °C at the beginning of the process, then decreasing as the process proceeds, with the final temperature system at 1250–1280 °C. Drawing process parameters: the heating temperature is controlled within the range of 800–1000 °C, and the drawing speed is contained within 4–10 m/min. A cathode (Institute of Metal Research, Chinese Academy of Sciences, Shenyang, China) used in magnetron with a diameter of 0.5 mm was selected for the experiment, in which the content of thorium oxide was 2 wt%.

The sample was heated in a vacuum high temperature induction furnace (Institute of Metal Research, Chinese Academy of Sciences, Shenyang, China) with a vacuum of 10^−3^ Pa. To simulate the working conditions of the cathode, the cathode samples were heated at three temperatures of 500 °C, 1100 °C, and 1300 °C in a high vacuum to observe the surface structure and valence characteristics, and to explore the formation and evolution of the cathode surface active layer. When the sample was heated to the preset temperature, to stabilize the surface composition of the piece, the heating device parameters were maintained for 60 min to keep the preset temperature.

Scanning electron microscope (SEM) (Institute of Metal Research, Chinese Academy of Sciences, Shenyang, China) and transmission electron microscope (TEM) (Institute of Metal Research, Chinese Academy of Sciences, Shenyang, China) were used to observe the surface topography. After cutting and grinding and polishing using wire cutting, and by mechanical plus manual grinding, the samples were ground from 200 μm to 30 μm in thickness. This is followed by pitting and using ion thinning to reduce the thin area to obtain a transmission sample. X-ray photoelectron spectrometer (XPS) (Institute of Metal Research, Chinese Academy of Sciences, Shenyang, China) was used to test and analyze the valence state change trend and content of the main elements on the surface of the samples treated at different temperatures, and to explore the role of thorium oxide in the cathode electron emission process [16]. XPS spectra were recorded using a monochromated Al kα (1486.6 eV) X-ray source and a hemispherical analyzer. The operating parameters were as follows: system base pressure- < 5 × 10^−10^ mbar, diameter of the X-ray beam-500 μm, take-off angle-45°. The high-resolution W 4f, O1s, and Th 3 d spectra were taken at pass energy of 20 eV and scan step of 0.05 eV; dwell time was 100 ms for each point.

## 3. Results and Discussion

### 3.1. Original Microstructure of Wire

**Figure 1:**
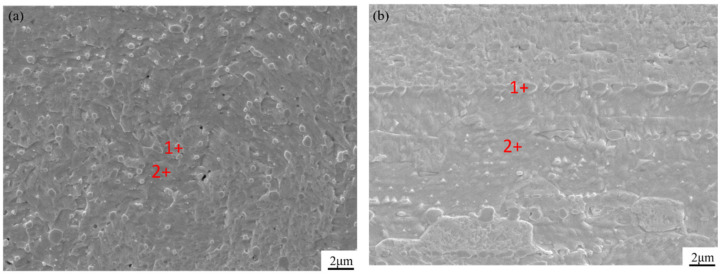
The morphology of a thoriated tungsten alloy coil (**a**) cross sectional (**b**) longitudinal section.

Figure 1a shows the cross-sectional morphology of a thoriated tungsten alloy coil with a wire diameter of 0.5 mm. The surface of the sample is relatively flat, and the granular material in the picture is thorium oxide particles with a size of about 0.5–1 μm, which are evenly distributed in the matrix. The longitudinal section morphology is shown in Figure 1b. Observed from the morphology, it is found that the structure is similar to the layered structure, and the surface of these layered structures and the gaps in the middle are mixed with granular substances. The quantitative analysis is shown in Table 1. The thorium content at position 1 is significantly higher than that at position 2, indicating a segregation of thorium at position 1. Part of the oxygen comes from thorium oxide, and part of it comes from tungsten oxide that has not been completely reduced. As a matrix element, tungsten has the highest content. It can be determined by energy spectrum analysis that the dark particles at position 1 are thorium oxide particles.

**Figure 2:**
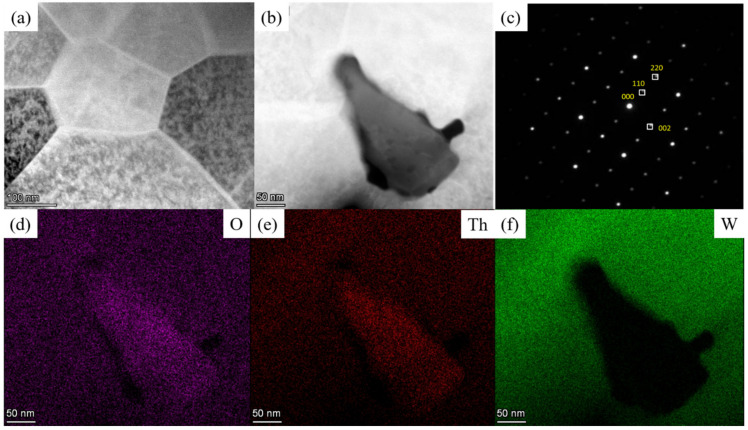
Microstructure of thoriated tungsten cathode: (**a**) Tungsten matrix; (**b**) ThO_2_ Organization; (**c**) Electron diffraction spots; (**d**–**f**) O, Th, W side scan analysis.

The microstructure of the thoriated tungsten cathode was studied using transmission electron microscopy (TEM). Figure 2a,b shows the microstructure of the tungsten substrate, and the tungsten grains are tightly bound, as can be seen in the TEM images. The presence of dark granular organization at the grain boundaries was analyzed by electron diffraction calibration and elemental face sweep, as shown in Figure 2c–f. The crystal structure can be identified as ThO_2_. The morphology is shown in Figure 2b.

### 3.2. The Morphology and Structure of the Surface Active Layer

For the thoriated tungsten cathode material studied in this article, the surface of the material may be enriched with nano-particles (thin film) under normal working conditions. The morphological structure and composition distribution of the active layer on the cathode surface would affect the electron emission capability of the cathode [19]. Significant electron emission would occur when a specific temperature was reached.

**Figure 3:**
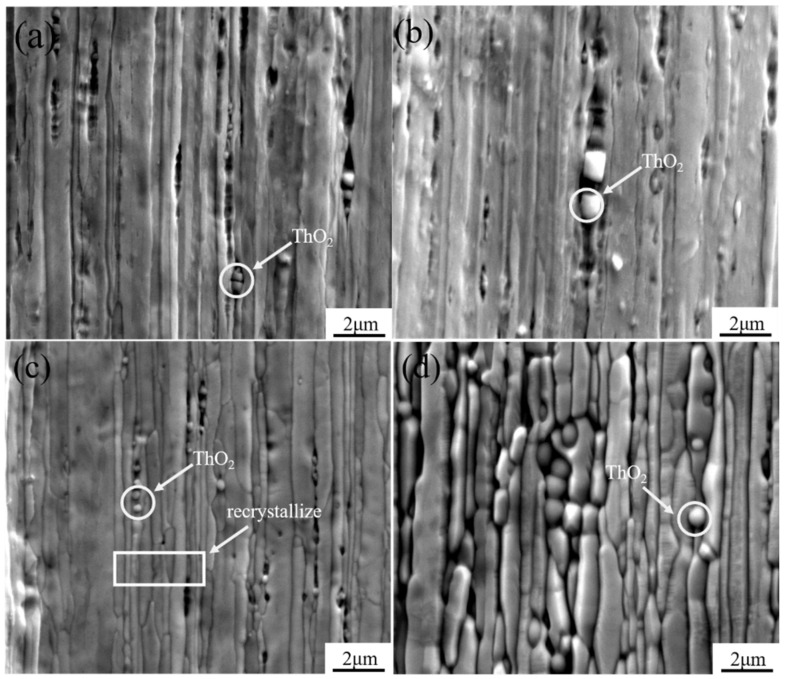
Surface morphology of samples at different temperatures: (**a**) room temperature; (**b**) 500 ℃; (**c**) 1100 ℃; (**d**) 1300 ℃.

**Figure 4:**
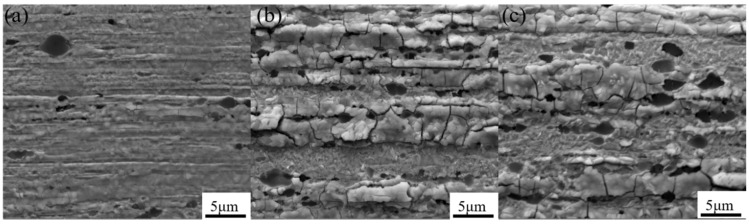
The longitudinal cross-sectional morphology of the cathode at different treatment temperatures: (**a**) 500 ℃; (**b**) 1100 ℃; (**c**) 1300 ℃.

Figure 3 shows the surface morphology of samples at different temperatures. It was found that the morphology gradually changed during the healing process. The matrix had a lamellar structure at low temperatures, and the surface was relatively flat. Part of the matrix began to recrystallize at 1100 °C, and completely recrystallized at 1300 °C, forming an active layer on the surface. Figure 4 shows the longitudinal cross-sectional morphology of the cathode at different treatment temperatures. It can be seen from the figure that as the heating temperature increases, diffusion channels appear inside the cathode. The fine crystal microstructure accelerated the diffusion of active elements from the body to the surface, so that the active elements quickly reached a new balance on the surface. The morphology of thorium oxide also changed during the healing process, from the initial cubic shape to a spherical shape. The reason was that the of surface thorium oxide evaporated during the heating process, and the spherical surface had the lowest free energy and the least consumption.

**Figure 5:**
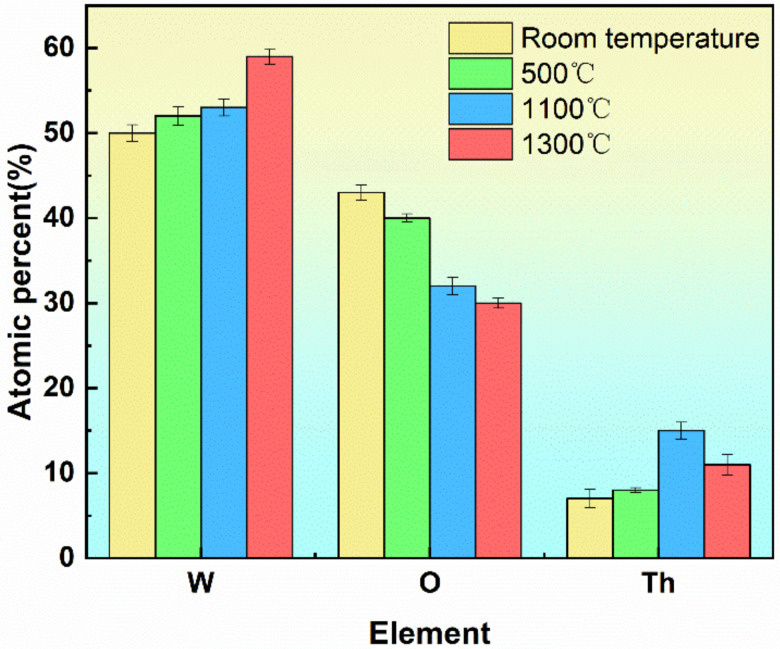
Changes of Th-W cathode composition at different temperatures.

The survey spectrum of each temperature was acquired by XPS to obtain the composition changes of the Th-W cathodes, as shown in Figure 5.

The results showed that the W content on the sample surface changed from 50% to 59%. At the same time, the thorium content first increased and then decreased. The range of the O changed from 43% to 30%. The surface thorium content was low at hypothermia. This is because some gases and impurities are still adsorbed on the surface of the sample brought into the vacuum from the atmosphere, affecting the experiment results. After the temperature rise, a part of the gas impurities adsorbed on the surface left the surface. The surface thorium content increased, and the active layer of thorium oxide on the cathode surface was gradually formed. The production of the active layer reduced the work function of the cathode surface, promoted electron emission, and improved the electron emission performance of the cathode surface. After the sample was heated to the preset temperature, it maintained for 60 min. Although the model had been at this temperature for a long time, the cathode surface active layer reached a relatively stable state. Even if the surface bared loss to some degree, it could still rely on the migration of the active material to the surface to supplement the active material, and maintain a certain dynamic material content on the cathode surface. It was beneficial to the electron emission of the cathode and to maintain better electron emission performance. As the heating temperature of the sample continued to increase, the surface thorium content decreased, and the cathode surface active elements began to lose. The consumption rate of surface-active material was higher than that of replenishment from the material to the surface. This would lead to a decline in electron emission performance and reduce the service life of the cathode.

**Figure 6:**
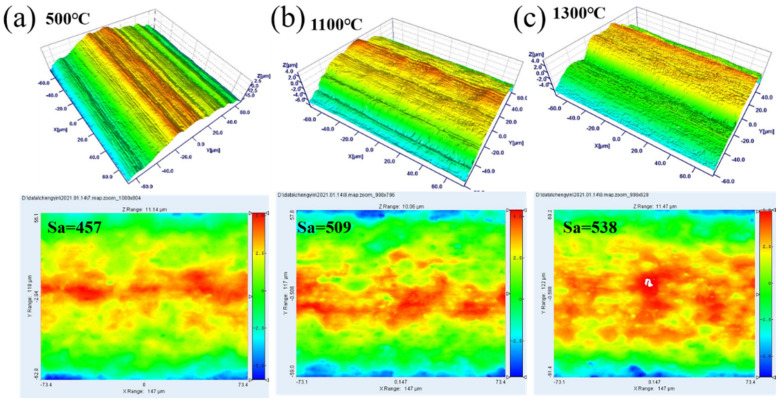
White light interference three-dimensional selected-area imaging. (**a**) 500 ℃; (**b**) 1100 ℃; (**c**) 1300 ℃.

To visually display the morphological characteristics of the cathode surface active layer, white light interference imaging was used to observe the morphological changes of the tungsten matrix surface under high temperature. Since gas impurities were adsorbed on the surface of the unheated samples, which affected the analysis result, samples with heating temperatures of 500 °C, 1100 °C, and 1300 °C were selected for observation. At 500 °C, due to the low processing temperature, only part of the adsorbed substances detached from the surface during this heating stage. The material itself did not undergo substance loss or migration, and the surface state was close to the original surface. The surface of the tungsten substrate had distinctive morphology features, with large protrusions in some areas and no continuous and regular changes in the morphology. Figure 6b,c were the three-dimensional images of white light interference at 1100 °C and 1300 °C. It could be seen that the surface of the tungsten substrate on which the surface active layer had been formed showed a continuous and regular up-and-down morphology. Such a topographic structure significantly increased the surface area, thereby increasing the electron emission center on the cathode surface, conducive to electron emission. The figure showed the surface roughness at three temperatures. It could be seen that as the temperature increased, the surface roughness increased, which was also consistent with the results of the previous scanning and white light interference.

**Figure 7:**
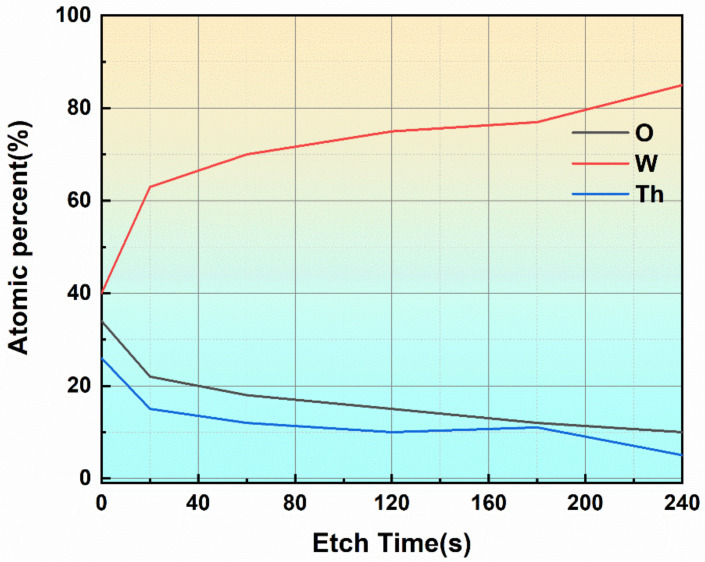
Etching area and composition change.

It has been known from the previous analysis results that at a higher heating temperature, an active layer composed of thorium oxide was formed on the surface of the tungsten substrate. This active layer played a crucial role in electron emission. Therefore, the surface of the tungsten substrate was deeply analyzed to obtain the distribution characteristics of the active layer along the depth direction. The analysis object was vacuum heated sample at 1300 °C, and an active layer had been formed on the surface of the sample. A flat area was selected on the surface of the tungsten substrate, and the Ar^+^ gun that came with the X-ray photoelectron spectrometer was used to etch the area chosen to analyze the composition information of the sample surface before and after the etching and the change of the content of each element with the etching time [20]. The etching time was shown on the abscissa, and the atomic percentage content was shown on the ordinate. The changes of various elements on the surface before and after etching with the etching time are shown in Figure 7.

The main constituent elements on the surface of the tungsten substrate before etching were Th, W, and O. In addition to the tungsten element in the substrate, oxygen, and thorium were mainly derived from the thorium oxide in the active layer. After the etching was completed, the surface of the sample was almost entirely composed of tungsten. It indicated that the etching effect of the Ar+ gun removed the active layer on the surface of the tungsten substrate. The surface of the sample that lost the active layer would be entirely composed of metallic tungsten. Such a composition of the surface could not achieve the effect of reducing the work function, and went against the effect of electron emission. It could also be seen that a large amount of thorium oxide on the surface of the tungsten substrate was concentrated and diffused by oxides, not the tungsten substrate itself. Within 1 min of the beginning of etching, the content of each element changed significantly, mainly as the content of thorium and oxygen dropped sharply, and correspondingly, the content of tungsten that constituted the matrix gradually increased. After etching more than 1 min, the change rate of the content of each element tended to be flat, indicating that the composition of the sample surface was basically stable at this time.

**Figure 8:**
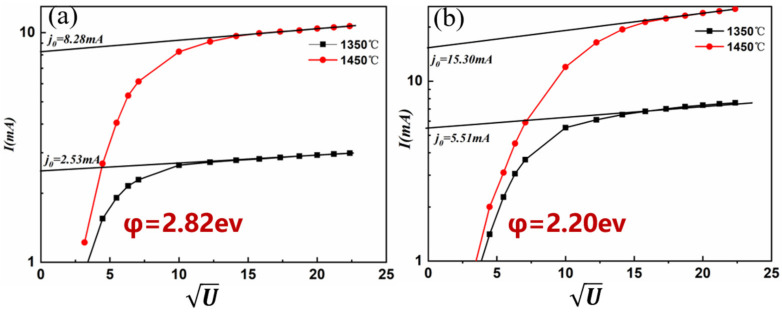
The zero-field current density and work function: (**a**) 500 °C, (**b**) 1300 °C.

Then, the zero-field current emission density and work function were measured using cathode emission performance test benches, and the results are shown in Figure 8.

It can be seen that the zero-field current emission density (j_0_) of the cathode increased with the increase of temperature. The calculation of the work function using the Richardson linear method is primarily based on the zero-field hot electron emission equation, as given below:

ln(j_0_T^−1^) = lnA − 11605φ_0_ T^−1^ (1)

where j_0_ refers to the zero-field emission current density of the material at a certain operating temperature (T), A denotes the electron emission constant and φ_0_ represents the work function. First, the obtained data were linearly fitted and the line slope was used to calculate the work function. One should note that the work function of the alloy decreased with the increase of temperature, indicating that less energy is required for the electrons to escape from the metal surface. Hence, electrons are easily emitted into the vacuum.

### 3.3. Study on the Valence State of Surface-Active Layer

The changing of 4f orbital of W(W4f), 1s orbital of O O1s and 4f orbital of Th Th4f at different temperatures was shown in Figure 8. The oxidation state W peak appeared on the surface of the sample. The oxidation state of W was caused by the fact that a small part of W had not been completely reduced during the preparation process of the cathode product. As the heating temperature increased, the W content in the oxidation state gradually decreased. The spectrum showed that the areas of the two peaks at 37.24 eV and 35.21 eV gradually reduced. As the heating temperature increased, the adsorbed oxygen content gradually reduced. This resulted from the adsorbed gas leaving the surface and being drawn from the vacuum chamber. Thorium existed on the surface of the cathode in the form of thorium oxide. As the heating temperature increased, the surface thorium oxide content increased, forming an active layer and improving the electron emission efficiency.

**Figure 9:**
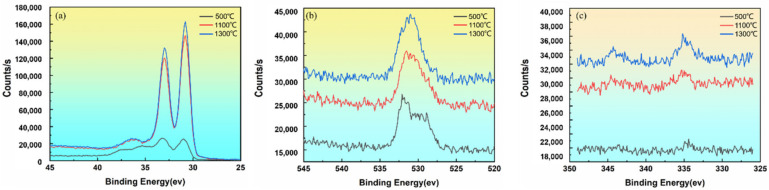
The change trend of the peak value at different temperatures: (**a**) W; (**b**) O; (**c**)Th.

**Figure 10:**
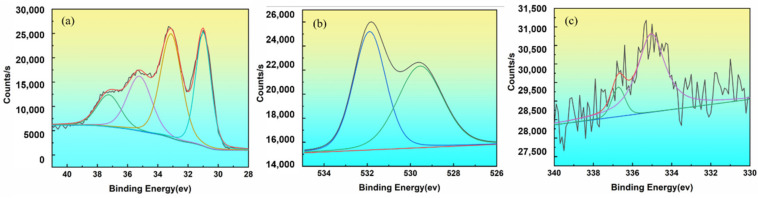
Fitting results peak on 1300 °C sample surface: (**a**) W; (**b**) O; (**c**)Th.

According to the fitting of the W4f peak at hypothermia (Figure 9a), the binding energy of the two W peaks was 37.24 eV and 35.21 eV, respectively, corresponding to W^6+^ and W^4+^. The fitting of the W4f, O1s and Th4f spectrum at 1300 °C was shown in Figure 10.

**Figure 11:**
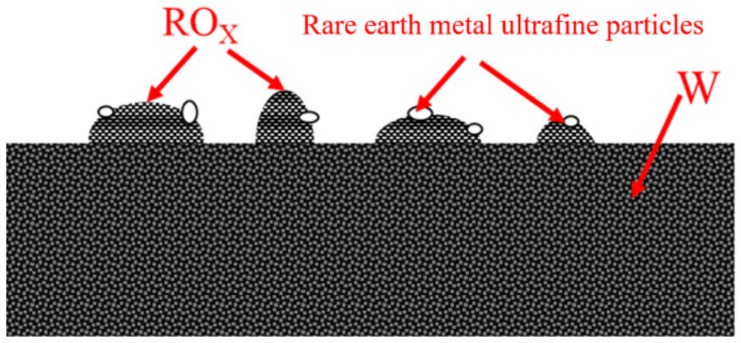
Metal ultrafine particles.

In addition to the oxidation state of tungsten, there were also peaks at 33.12 eV and 30.99 eV, which were the 4f5/2 and 4f7/2 peaks of metallic tungsten, respectively. The double peak spacing was 2.1, and the peak area ratio was close to 3:4, consistent with the peak position and peak shape characteristics of metallic tungsten. The peaks of the samples at low temperatures indicated that there were two main types of oxygen, namely 531.6 eV adsorbed oxygen and 529.8 eV lattice oxygen. The lattice oxygen was derived from W oxide and oxide [21,22,23,24]. According to the fitting of the Th4f peak at 1300 °C (Figure 10c). The decrease in oxygen content indicated an active layer formed by an oxygen-deficient Th-O compound on the surface of the thoriated tungsten cathode. Rare earth metal ultrafine particles were produced at high temperatures, as was shown in Figure 11.

These nano-sized metal ultrafine particles were adsorbed on oxides to form atomic groups, which increased the concentration of high-energy valence electrons. The proper coordination of the elements in the atomic group made the entire nuclear force field small. The high-energy valence electrons of the metal ultrafine particles were efficiently emitted into the vacuum, which was a direct contributor to thermionic emission. The relationship between the work function of the atomic group and the work function of the block was [25]:

(2) $$\emptyset_{c}-\emptyset_{b}=-\frac{2M}{ZC_{F}m}\times \frac{F}{r}+\frac{3e^{2}}{8r}$$

In this formula, $\emptyset_{c}$ represented the work function of the atomic group,$\;\emptyset_{b}$ the work function of the bulk metal, M the mol mass, Z the number of charged electrons, C the Faraday constant, m the mass of the atomic group, F the surface free energy of the atomic group, r the radius of the atomic group, and $\frac{3e^{2}}{8r}$ the effect of the mirror image force. It could be seen from the formula that as the size of the atomic group decreased, the work function of the atomic group decreased. The above conclusion could be applied to the metal ultrafine particles in the active oxygen layer on the cathode surface. That is, the work function of the metal ultrafine particles reduced compared with that of the metal. An active layer constituted a good electron emitter and reduced the work function. Therefore, the high-energy valence electrons of metal ultrafine particles were more likely to be emitted into the vacuum.

## 4. Conclusions

1. Under the action of vacuum and high temperature, the tungsten matrix recrystallises, the grain boundaries increase and the active atoms migrate outwards along the grain boundaries, which facilitates the formation of the active layer.
2. For surfaces where an active layer has formed, the etching action of the Ar+ lance removes the active layer from the surface of the tungsten substrate. After the surface oxides and active elements have been removed, the surface is at this point almost composed of tungsten and such a surface does not have good electron emission capability.
3. The active layer formed by the thorium-oxygen compound at high temperature would produce metal ultrafine particles, which increase the concentration of high-energy valence electrons, reduce the electron work function, and improve the emission capability.

## Figures and Tables

**Table 1:** Element quantitative analysis (wt%).

Element	Test Position	Cross-Sectional	Average Value	Longitudinal Section	Average Value
Th	1	30.76	31.87	26.21	24.35
		32		23.8	
		33.35		23.04	
	2	15.91	16.82	4.63	5.21
		17.1		6.1	
		18.17		4.9	
W	1	54.15	53.26	45.47	46.26
		53.8		47.1	
		52.33		46.21	
	2	76.13	75.31	60.06	59.66
		75.2		58.7	
		74.6		60.22	
O	1	17.09	14.87	28.32	29.39
		15.2		29.1	
		13.32		30.75	
	2	9.79	7.87	35.3	35.13
		7.7		35.2	
		6.12		34.89	

## Data Availability

The data presented in this study are available on request from the corresponding author.
